# Study of community-living Alzheimer’s patients’ adherence to the Mediterranean diet and risks of malnutrition at different disease stages

**DOI:** 10.7717/peerj.5150

**Published:** 2018-07-06

**Authors:** Mariona Rocaspana-García, Joan Blanco-Blanco, Alfonso Arias-Pastor, Montserrat Gea-Sánchez, Gerard Piñol-Ripoll

**Affiliations:** 1 University of Lleida, Faculty of Nursing and Physiotherapy, GRECS-IRBLleida, Lleida, Spain; 2 GESEC Research Group, Faculty of Nursing and Physiotherapy, University of Lleida, Health Care Research Group (GRECS), Biomedical Research Institute of Lleida (IRBLleida), Lleida, Spain; 3 Unit of Cognitive Disorders, Santa Maria University Hospital, Clinical Neuroscience Research, Biomedical Research Institute of Lleida (IRBLleida), Lleida, Spain

**Keywords:** Alzheimer’s disease, Mediterranian diet, Nutrition Assessment, Nutritional Status, Cognition

## Abstract

**Background:**

Alzheimer’s disease (AD) is a neurodegenerative disease that is characterized by deficits in episodic memory. It is the most common form of dementia and affects 50–70% of patients with cognitive impairments over the age of 65. Elderly people are particularly susceptible to malnutrition and that risk is even higher in patients with AD. This study assessed the nutritional status of patients with AD at different stages of AD and explored how that status correlated with cognitive, functional and behavioural variables and caregiver overburden. The characteristics of the diet and the degree of adherence to the Mediterranean diet were also analysed.

**Methods:**

This was a cross-sectional study that was representative of the general population and it was carried out in the Unit of Cognitive Disorders, Lleida, Spain. The participants were 111 subjects with AD who were aged 65 and over and still living at home. The subjects’ nutritional status was assessed using the Mini Nutritional Assessment (MNA) and Controlling Nutritional Status system. The monthly food intake was estimated using the short Food Frequency Questionnaire and adherence to the Mediterranean diet was evaluated using the Mediterranean Diet Score. The Mini Mental State Examination (MMSE), Global Deterioration Scale, Neuropsychiatric Inventory (NPI) and Zarit Burden Interview (ZBI) were also used.

**Results:**

We found that 68% of the subjects presented with a risk of malnutrition and 19% were malnourished according to the MNA scale. Patients ate a higher quantity of meat and dairy products than recommended and fewer products from the remaining healthier food groups. Of the 111 subjects, 73% showed low adherence to the Mediterranean diet and 27% showed moderate adherence. There was a partial correlation between nutritional status and the MMSE when the data were adjusted for age and sex (*r* = 0, 318; *p* = 0.001) and inverse correlations were found for functional status (*r* =  − 0.283; *p* = 0.004) and the NPI (*r* =  − 0.409; *p* = 0.000) and ZBI scales (*r* =  − 0.433; *p* = 0.000) when they were adjusted using the same variables. The ZBI scale (OR 1.08, 95% CI [1.01–1.15]) showed an increase in the risk of malnutrition in the multivariate analysis.

**Discussion:**

Alterations in nutritional status were more common during the advanced stages of AD and were also associated with behavioural changes and caregiver overburden. Low adherence to traditional healthy diets in Mediterranean countries and food intake profiles should be considered when managing patients with AD. Other countries can use the results to examine diets in people with AD that are high in meat and dairy and low in healthy food groups like fruit and vegetables.

## Introduction

Alzheimer’s Disease (AD) is a neurodegenerative disease that is characterised by episodic memory loss and alterations in other cognitive domains, such as language, praxia and executive functions. Patients also display a great variety of psychological and behavioural symptoms, such as apathy, depression and hallucinations (McKhann et al., 2011). AD is the most frequent form of dementia and it accounts for 50–70% of patients with cognitive impairment over the age of 65 (Winblad et al., 2016). Studies have shown that the incidence of AD increases exponentially with age, with a prevalence of between 6–8% at 65 years of age and 30% from the age of 85 (Fratiglioni & Qiu, 2011; Prince et al., 2013).

Although its aetiology is not precisely known, amyloid accumulation is the most frequent and specific pathological alteration of the disease and this plays a role in the development of all the physiopathological process that are triggered during AD. However, there are multiple factors that can promote, or enhance, the development of the disease and these include female sex, age and all the cardiovascular risk factors, including high blood pressure, diabetes, insulin resistance, hypercholesterolemia, obesity, smoking, physical exercise and diet (Kivipelto et al., 2005; Beydoun, Beydoun & Wang, 2008; Peters et al., 2008; Craft, 2009; Etgen et al., 2011; Candeias et al., 2017; Gottesman et al., 2017). Studies have indicated that patients with AD present with weight loss years before the onset of the disease (White, McConnell & Bales, 2004; Knopman et al., 2007; Gao et al., 2011). Once the disease has started to develop, people with AD have problems related to buying food and preparing meals and even remembering whether or not they have eaten. When AD reaches the more advanced stages, patients need help to eat, because they experience eating behaviour disorders, such as difficulties swallowing and, or, chewing (Doty, 2001; Luchsinger & Mayeux, 2004; Shatenstein, Kergoat & Reid, 2007). All these factors may have an impact on the patients’ nutritional status.

Even in developed societies, elderly people are particularly susceptible to the risk of malnutrition (Saka et al., 2010) and this risk is even higher in patients with AD, where the prevalence of malnutrition ranges between 14–41% (Cuervo et al., 2009; Roque, Salva & Vellas, 2013; Droogsma et al., 2013). Several studies have proved that malnutrition, weight loss and cachexia are associated with cognitive dysfunction, a decrease in an individual’s capacity to carry out daily activities and an increase in morbidity and mortality, (Guerin et al., 2005a; Droogsma, Van Asselt & De Deyn, 2015). Therefore, an appropriate nutritional intervention is vital if the disease is to be correctly managed (Scarmeas et al., 2009a; Scarmeas et al., 2009b). The Mediterranean diet provides key elements for the maintenance of cerebral structures and functions, such as omega-3 fatty acids, folic acid, carotenoids and vitamin E (Serra et al., 2003; Garcia, Berenguer & González, 2006; Giuseppe et al., 2013). Although it varies by country and regions, the Mediterranean diet is high in vegetables, fruits, legumes, nuts, beans, cereals, grains, fish and unsaturated fats, such as olive oil. In contrast, it usually includes a low intake of meat and dairy foods.

However, studies have also shown that this diet is increasingly difficult to adhere to, even in Mediterranean countries, due to social and occupational changes as people move around and enjoy more varied cuisines. These difficulties are enhanced in patients with AD, as they find it hard to prepare their own meals (Abellán et al., 2016).

In view of these existing issues, many questions have remained unresolved with regard to the relationship between nutrition and cognition in AD patients. The purpose of this study was to: (i) evaluate patients’ nutritional status at different stages of AD, (ii) evaluate their adherence to the Mediterranean diet and (iii), correlate the links between nutritional status and several aspects of AD, such as cognition, behavioural symptoms, functionality and the caregivers’ burden.

## Materials and Methods

### Ethical standards

This study was conducted according to the guidelines laid down in the Declaration of Helsinki and all procedures involving human subjects were approved by the medical ethics committee of the Hospital Universitario de Santa María in Lleida (ref number 1276). All patients and caregivers provided written, informed consent.

### Study design and population

This was an observational study that prospectively and consecutively recruited 111 subjects who lived in their own homes and attended the Cognitive Disorders Unit at the Hospital Universitario de Santa María in Lleida. All patient follow ups in this Unit and all diagnoses of AD followed the criteria developed by McKhann et al. (2011).

The patients in the study had all undergone neuropsychological evaluation and structural neuroimaging, namely computed tomography and, or, magnetic resonance imaging, at the discretion of the specialist in charge. The analytical protocol included a blood count and basic biochemistry, thyroid-stimulating hormone, vitamin B12, folic acid and syphilis serology.

Patients were included if they were 65 years or older, had been followed up in the Unit when they were diagnosed with AD and had a Global Deterioration Scale (GDS) score of 3–6. We excluded patients who had been diagnosed with any dementia other than AD or who had any somatic, psychiatric, or neurological disorders that might have caused their cognitive impairment. Patients were also excluded if they had other chronic diseases that could contribute to the alteration of nutritional status independently of AD, such as chronic kidney disease, severe heart failure or cancer, and patients with any visual or auditory impairment that meant they would not be able to perform the tests included in the study. The other exclusion criteria were patients who lived in long-term care facilities, those who did not have anyone who could provide reliable information on their eating habits, and those whose had mobility problems that meant that anthropometric measures could not be taken.

### Assessment instruments and data collection

A trained nurse carried out the structured interviews for data collection from January 2014 to January 2016. The epidemiological data including age, gender, education, family history and a medical history of hypertension, type 1 and 2 diabetes, hypercholesterolemia and depression. We also reviewed their smoking and alcohol habits. Habitual smoking was defined as 10 or more cigarettes per day and the patient was at risk of alcoholism if their alcohol intake was more than 20–30 grams per day. The time it took for their symptoms to evolve was also recorded.

Cognitive functions were examined using the Mini Mental State Examination (MMSE) (Folstein, Folstein & Mchugh, 1975) and functionality was evaluated using the Global Deterioration Scale (GDS), which is broken down into seven different stages: 1–2 are considered to be the pre-dementia stages and 3–7 cover the continuum from mild cognitive impairment to dementia (Reisberg et al., 1982). Patients who were at stage seven were excluded, as they rarely attended hospital outpatient clinics.

Neuropsychiatric symptoms were evaluated using the Neuropsychiatric Inventory (NPI) (Cummings et al., 1994; Vilalta et al., 1999), which is the most widely used scale for determining the frequency and intensity of neuropsychiatric disorders in AD patients. It consists of 12 items: delusions, hallucinations, agitation/aggression, dysphoria, anxiety, euphoria, apathy, disinhibition, irritability/lability, aberrant motor activity, night-time behavioural disturbances and appetite/eating disorders. The total value is obtained by multiplying the frequency by the severity. Frequency is assessed using a scale of 0–4 where zero is absent, one is occasionally, namely less than once per week, two is often, which is about once per week, three is frequently, which is several times per week and four is very frequently, namely daily or continuously. Severity is measured on a 1–3 scale, where one is mild, two is moderate and three is severe.. The caregiver is asked whether the patient’s behaviour has changed since the onset of dementia and, if so, whether the altered behaviour was present during the month before the NPI was used. The total score is the sum of all the subscale scores.

Finally, the burden of caregivers was evaluated using the Zarit Burden Interview (ZBI) (Martín et al., 1996), which is a well-known caregivers’ self-report measure that has been used in many dementia studies. The revised version contains 22 statements that the caregiver is asked to assess by using a five-point scale, with response options ranging from one to never to five for nearly always. Higher total scores indicate higher caregiver burdens.

All patients were examined to obtain the following anthropometric parameters: height, weight, body mass index (BMI), waist circumference, mid-upper arm circumference, calf circumference and triceps skin-fold measurements.

The patients’ nutritional status was assessed using the Mini Nutritional Assessment (MNA) scale (Guigoz, Vellas & Garry, 1994), the short Food Frequency Questionnaire (FFQ) (Trinidad et al., 2008) and the CONtrolling NUTritional status (CONUT) system (Ulíbarri et al., 2005).

The MNA consists of 18 questions divided into four nutritional areas. The four anthropometric measurements are BMI, mid-upper arm circumference, calf circumference and weight loss and the global assessment comprises six questions related to lifestyle, medication and physical and mental state. The dietary assessment comprises six questions related to daily food intake, intake problems and a subjective assessment and there is also a question on the patient’s self-perception of sufficient intake and a question on self-perception of their general health. The maximum score is 30: a score of less than 17 is considered malnutrition, a score of 17–23.5 is considered at risk of malnutrition and scoring 24 or higher is considered a satisfactory nutritional status (Guigoz, Vellas & Garry, 1994).

The short FFQ consists of 45 items that asks the patient or caregiver for details of the weekly or monthly intake of certain foods (Trinidad et al., 2008).

The CONUT System of Nutritional Control is used to alert medical staff that a patient has malnutrition and it consists of three parameters: two biochemical parameters (serum albumin and cholesterol) and an immunological parameter (total lymphocytes). The maximum score is 12, with 0–4 indicating low undernourishment, 5–8 moderate undernourishment and 9–12 severe undernourishment (Ulíbarri et al., 2005).

Adherence to traditional Spanish diet was measured using the Mediterranean Diet Score (MDS), which assesses whether it is low (scores 0–3), moderate (scores 4–5) or high (scores 6–8). The score covers the daily intake, in grams, of different food groups: vegetables, fruit-nuts, pulses, fish, alcohol, cereal, meat and dairy products. A score of one indicates that the intake is higher than recommended and a score of zero indicates that intake is lower than recommended. High scores for meat and dairy products were regarded as harmful, but they were regarded good for other food groups. (Trichopoulou et al., 1995; Hu et al., 2002; Trichopoulou et al., 2003). We also took into account whether the patient ate alone or with others, whether they had chewing difficulties, whether they prepared their own meals and the patient’s and caregiver’s levels of income. These aspects were not included in the validated scales, but scientific literature has shown them to be relevant to the patients’ nutritional status (Grundman et al., 1996; Riviere et al., 1999). These additional assessments were recorded during a semi-structured interview and the data were dichotomized as yes or no.

The Biomedical Ethics Committee of the Lleida country approved the study and all patients and caregivers provided written informed consent.

### Statistical analysis

The statistical data analyses were performed using SPSS for Windows, version 16, (SPSS Inc, Chicago, IL, USA). The results of the different variables were expressed as their means and standard deviations for the quantitative variables and as percentages for the qualitative variables. The statistical significance of the differences between the quantitative and qualitative variables were assessed using analysis of variance (ANOVA) and Pearson’s chi-square test was used to compare the percentages of the variables. Spearman’s bivariate correlation, adjusted by sex and age, was used to investigate the existence of correlations between the clinical variables. Multivariate logistic regression models were built for predicting malnutrition and the risk of malnutrition. Both models were built with variable selections by backwards stepwise procedures based on likelihood ratios. Values of less than 0.05 were considered statistically significant.

## Results

We studied 111 patients (63.4% women) with an average age of 78.5 ± 6.4 years. With regard to their family history, 38.4% presented with first degree AD and all experienced onset when they were more than 65 years of age. In terms of pathological background, the most prevalent risk factor was arterial hypertension (58.7%), followed by dyslipidemia (46.8%) and type 1 and 2 diabetes mellitus (19.3%) (Table 1). We noted that 45% of the patients were overweight, only 3.6% had been habitual smokers and 27.9% had consumed levels of alcohol that put them at risk for alcoholism.

**Table 1:** Socio-demographic, cognitive and behavioural characteristics and caregiver burden of the study population, and nutritional status according to MNA scale.

Variable	Normal (*n* = 15)	Risk of malnutrition (*n* = 75)	Malnutrition (*n* = 21)	*p* value
Age	79.0 ± 5.6	77.7 ± 6.4	81.0 ± 6.1	0.96
Women (%)	53.3%	62.7%	76.2%	0.34
HTA	73.3%	58.9%	50.0%	0.38
DM	13.3%	25.1%	40.0%	0.03
Hypercol	46.7%	47.9%	45.0%	0.97
Depression	26.7%	31.9%	57.1%	0.07
BMI	27.2 ± 3.8	27.4 ± 4.1	24.6 ± 3.3	0.01
Waist	98.4 ± 11.2	98.9 ± 10.7	95.3 ± 9.8	0.39
Mid-upper arm	28.9 ± 6.2	29.6 ± 3.3	28.2 ± 3.6	0.18
Skin fold	13.5 ± 5.3	16.2 ± 6.0	18.3 ± 5.0	0.19
Albumin	4.2 ± 0.06	4.1 ± 0.09	4.1 ± 0.44	0.81
Total cholesterol	203.2 ± 49.6	198.0 ± 38.7	199.5 ± 39.2	0.59
LDL	114.2 ± 21.4	118.5 ± 31.9	126.7 ± 28.9	0.18
MMSE	17.1 ± 6.3	19.8 ± 5.2	14.5 ± 7.0	0.03
GDS	4.0 ± 1.0	3.9 ± 0.8	4.5 ± 1.2	0.12
Zarit	37.2 ± 15.8	48.4 ± 16.2	50.3 ± 12.9	0.05
NPI	9 ± 8.5	11.5 ± 11.3	18.8 ± 21.9	0.06

**Notes.**

TITLE TSH Thyroid-stimulating hormone HTA Arterial hypertension DM Diabetes mellitus types 1 and type 2 Hypercol Hypercholesterolemia BMI Body mass index LDL Low density lLipoproteins MMSE Mini Mental State Examination GDS Global Deterioration Scale NPI Neuropsychiatric Inventory

The percentage of patients at risk of malnutrition was 67.6%, while 18.9% presented with malnutrition according to the MNA scale (Table 2). No differences were observed between the sexes.

**Table 2:** Nutritional characteristics of the study population according to the MNA and CONUT scales.

MNA	Men (%)	Women (%)	Total (%)	CONUT Low malnutrition alert (%)
Normal nutritional status	7 (17.5)	8 (11.3)	15 (13.5)	2 (6.7)
Risk of malnutrition	28 (70.0)	47 (66.2)	75 (67.6)	22 (73.3)
Malnutrition	5 (12.5)	16 (22.5)	21 (18.9)	6 (20.0)
Total	40 (36.0)	71 (64.0)	111 (100)	30 (100)

A sub sample of 30 subjects was also assessed using the CONUT scale. All subjects received a low malnutrition alert, although 73.3% of the total sample were at risk of malnutrition according to the MNA assessment. We stopped using it the CONUT scale because of its reduced ability to classify patients.

Despite the high scores for the patients’ malnutrition risk, the BMI analysis revealed that 45% were overweight and 18.9% were obese, meaning that 63.9% of subjects analysed were above the BMI for normal weight. Another highlight is 69.3% of patients with risk of malnutrition were obese or overweight, so it seems that BMI is not a good indicator in this population.

The short FFQ questionnaire was used build up a more detailed picture of the intake of the different food groups. As a general rule, 85.6% of patients consumed a lower quantity of vegetables than recommended and this was also the case for other good groups: 81.1% for fruit and nuts, 100% for pulses, 89.2% for cereals and 95.5% for fish. In contrast, almost half of the patients (46.8%) ate more meat than recommended and 84.7% consumed more dairy products (Trichopoulou et al., 1995) (Table 3). No significant differences were observed between the sexes or the different stages of the disease.

**Table 3:** Food intake evaluation of the study population, according to the FFQ scale.

	% less than recommended average			% more than recommended average		
	Men (%)	Women (%)	Total (%)	Men (%)	Women (%)	Total (%)
Vegetables	36 (90.0)	59 (83.1)	95 (85.7)	4 (10.0)	12 (16.9)	16 (14.4)
Fruit and nuts	37 (92.5)	53 (74.6)	90 (81.3)	3 (7.5)	18 (25.4)	21 (18.9)
Dairy products	8 (20.0)	9 (12.7)	17 (16.1)	32 (80.0)	62 (87.3)	94 (84.7)
Cereals	39 (97.5)	60 (84.5)	99 (89.3)	1 (2.5)	11 (15.5)	12 (10.8)
Pulses	40 (100)	71 (100)	111 (100)	0 (0)	0 (0)	0 (0)
Meat	30 (75.0)	29 (40.8)	59 (53.6)	10 (25.0)	42 (59.2)	52 (46.8)
Fish	35 (87.5)	71 (100)	106 (95.5)	5 (12.5)	0 (0)	5 (4.5)
Alcohol	24 (60.0)	56 (78.9)	80 (72.3)	16 (40.0)	15 (21.1)	31 (27.9)

When we assessed adherence to the Mediterranean diet using the MDS, we found that 73% of subjects demonstrated low adherence and 27% had moderate adherence (Table 4). It is worth noting that no patient met the criteria for good adherence to the Mediterranean diet and there were no significant differences between adherence to Mediterranean diet and the nutritional status, according to the MNA (*p* = 0.62).

**Table 4:** Adherence to the Mediterranean diet, according to the MDS

Adherence to DiMed	Men, *n* = 40 (%)	Women, *n* = 71 (%)	Total *n* = 111 (%)
Diet score (0–3) = Low	32 (80.0)	49 (69.0)	81 (73.0)
Diet score (4–5) = Moderate	8 (20.0)	22 (31.0)	30 (27.0)
Diet score (6–8) = High	0 (0.0)	0 (0.0)	0 (0.0)

Further data analysis showed a possible correlation between the patients’ nutritional status and their cognitive performance, functionality and degree of behavioural alterations. A partial correlation was found when the nutritional status was assessed with the MMSE (*r* = 0,318; *p* = 0.001), and adjusted for age and sex, and an inverse correlation was found when the functional situation was assessed with the GDS (*r* =  − 0.283; *p* = 0.004) on the same basis. In addition, a correlation was found with the NPI (*r* =  − 0.409; *p* = 0.000) and with the ZBI scale (*r* =  − 0.433; *p* = 0.000) (Table 5), adjusted for age and sex. In the multivariate analysis, the NPI score (*r* =  − 0.249; *p* = 0.047) (OR 0.99; 95% CI [0.92–1.07]) and the ZBI scale (*r* =  − 0.357; *p* = 0.004) (OR 1.08; 95% CI [1.01–1.15]) showed a higher risk of malnutrition controlled by the scores in the MMSE and the GDS.

**Table 5:** GDS, MMSE, NPI, Zarit and MNA correlations in patients with Alzheimer’s sdisease.

	MNA			
	Screening		Total	
	Spearman’s correlation (*r*)	*p* value	Spearman’s correlation (*r*)	*p* value
MMSE	0.283	0.000	.318	0.001
GDS	−0.298	0.001	−.283	0.004
NPI	−0.306	0.003	−.409	0.000
Zarit	−0.306	0.004	−.433	0.000

**Notes.**

TITLE MMSE Mini Mental State Examination GDS Global Deterioration Scale NPI Neuropsychiatric Inventory

Finally, patients’ eating habits that were not reflected in the scales we used, and those that were of potential interest, were assessed but none of these variables were significantly associated to a worse nutritional status (*p* > 0.05). These were whether the patient ate alone, needed help to eat, were able to prepare their own food, whether they had any chewing difficulties and the income levels of the patient and caregiver (Table 6).

**Table 6:** Correlation between eating difficulties and the caregiver’s income with the patients’ risk of malnutrition.

	MNA			
	Screening		Total	
	Pearson correlation (*r*)	*p* value	Pearson correlation (*r*)	*p* value
Eats in company	0.159	0.171	−0.059	0.171
Needs help to eat	0.225	0.051	−0.213	0.065
Prepares meals	−0.046	0.696	0.052	0.654
Has chewing difficulties	0.011	0.925	−0.045	0.704
Patient’s income	−0.091	0.448	−0.146	0.220
Caregiver’s income	−0.262	0.058	0.130	0.352

## Discussion

This study evaluated the nutritional status of a population of patients aged over 65, who were diagnosed with AD and living at home. Since recruitment was consecutive, and the unit where it took place was the only one in the health region, the sample obtained was representative of the region studied. It can be inferred from the socio demographic characteristics and the prevalence of cardiovascular risk factors that this population was similar to that of Spanish and European studies on populations diagnosed with AD (De Pedro-Cuesta et al., 2009; Warchol-Celinska et al., 2015; Niu et al., 2017).

When we used the MNA scale, the high prevalence of malnutrition (18.9%) and risk of malnutrition (67.6%) was notable, as it was even higher than expected for people of the same age with no cognitive impairment. The prevalence for the latter group has been reported to range from 15.2% and 50% according to different studies (Cuervo et al., 2008; Zekry et al., 2008; Jürschik et al., 2009; Unanue et al., 2009; De Luis et al., 2011; Nykänen et al., 2013). In similar studies carried out in the Mediterranean region, namely Italy and France, the prevalence of malnutrition and risk of malnutrition were 42.8% and 28% respectively, using the MNA scale (Ousset et al., 2008; Spaccavento et al., 2009). In a study carried out by Roque, Salva & Vellas (2013) on a population with the same characteristics as our study, 40.8% faced a risk of malnutrition, but only 4.8% actually presented with malnutrition. Finally, a literature review carried out by Guigoz (2006) on subjects diagnosed with AD found that the prevalence of the risk of malnutrition ranged between 19% and 36% and the actual presence of malnutrition ranged between 0% and 6% (Andrieu et al., 2001; Rivière et al., 2001; Nourhashemi et al., 2005; Gillette-Guyonnet et al., 2013).

The causes of the high prevalence of malnutrition may be secondary to multiple aspects, such as difficulties preparing meals and eating a varied diet, having swallowing problems or poor food absorption. We considered other parameters that were not included in the traditional scales that could be related to the degree of undernourishment, such as eating alone, needing help to eat or prepare meals and the patient’s and caregiver’s economic situation. However, the results did not show any statistically significant associations.

Several epidemiological studies have demonstrated that being overweight or obese in middle age may increase the risk of AD (Whitmer et al., 2005; Whitmer et al., 2007; Gustafson et al., 2012). By contrast, other studies have related losing weight to the development of cognitive impairment (Gillette-Guyonnet et al., 2007; Shatenstein, Kergoat & Reid, 2007). The anthropometric variables, including the BMI analysis, were no use in our study when it came to evaluating the patient’s nutritional status. The average BMI of the patients at risk of malnutrition (27.4 ± 4.1) indicated that a high number of patients were overweight. This could lead us to believe that the subjects in our study were undernourished due to an unhealthy diet and not because they didn’t eat enough food. This finding could be supported by the low ratio of MDS observed in our patients. Similar results were found by Roque, Salva & Vellas (2013) in their study, where the risk of malnutrition was not supported by other tools like the subject’s BMI. In line with our results, that study observed that the presence of malnutrition could coexist with a normal BMI or even a BMI that was higher than normal.

Regarding the use of other scales, we could not find studies that assessed the usefulness of the CONUT scale in populations diagnosed with AD, but our results showed that the MNA scale was more useful in evaluating nutritional status than the CONUT scale in this type of population.

It is notable from our results that a worse nutritional status correlated with worse cognitive, functional and behavioural performance and with greater caregiver overburden, when these factors were assessed using the ZBI scale. A possible explanation for this is that patients with more advanced stages of AD habitually present with higher risks of behavioural alterations, which can be associated with greater caregiver overburden. Some authors have associated the risk of malnutrition, both in cognitively healthy elderly people and those with dementia, to factors such as the presence of behavioural symptoms, namely delirium, hallucinations, apathy and depression (Guerin et al., 2005b; Spaccavento et al., 2009; Isaia et al., 2011) and worse scores with the MMSE (Nykänen et al., 2013; Roque, Salva & Vellas, 2013). This would help to explain the association between worse nutritional status and greater caregiver overburden in our sample, as observed in the literature (Roepke et al., 2011; Legido et al., 2013). For this reason, it is important to provide caregivers with support and advice on changes associated with AD, to help them deal with those changes in the best possible way and to reduce the levels of stress and overburden and ensure the person they care for has a better nutritional status.

Another result worth highlighting is that in the advanced stages of AD the patient’s nutritional status worsened, as found in previous studies (Botella & Ferrero, 2004; Luchsinger & Mayeux, 2004; Vieira, Figueiredo & Garcia, 2015).

Finally, with regard to the Mediterranean diet, Catalonia is considered one of the most important regions in the world for this diet, but a very high percentage of patients in our sample (73%) showed low adherence to this diet. In general, they had a higher intake of meat and dairy products than recommended and a lower intake of fruit and vegetables, especially pulses. However, these results were similar to those published by other recent studies on Spanish populations, including the study carried out by Abellán et al. (2016), who also observed low adherence to the Mediterranean diet by their general study population. This suggests that while other countries in the world are trying to adopt the Mediterranean diet as their reference diet, Spain is abandoning it. A progressive detachment from the Mediterranean diet pattern by the Spanish population, and an unbalanced diet, was observed in a study carried out by Ruiz et al. (2015), although low adherence to Mediterranean diet was not correlated with body weight. This trend was also observed in the present study.

Our study provides important data, since several studies have demonstrated that the Mediterranean diet has been associated with greater cognitive performance and greater cerebral volume (Titova et al., 2013). Gardener et al. (2012) studied elderly people and observed that subjects diagnosed with AD had a lower score when they followed a Mediterranean diet, followed by subjects with mild cognitive impairment. In contrast, subjects without cognitive impairment presented the highest scores for adherence to the Mediterranean diet. This study also observed a correlation between the baseline Mediterranean diet score and the change in the MMSE score between the baseline assessment and the 18-month follow up of healthy subjects (Gardener et al., 2012). Several meta-analyses have suggested that greater adherence to a Mediterranean diet have been associated with lower cognitive deterioration, dementia and AD (Van de Rest et al., 2015) and even lowered the risk of progression of mild cognitive impairment into AD (Scarmeas et al., 2009a; Scarmeas et al., 2009b; Singh et al., 2014). Sofi et al. (2010) observed that a two percentage point increase in adherence to the Mediterranean diet score decreased the risk of the incidence of neurodegenerative diseases (risk ratio 0.87; 95% CI 0.81, 0.94). Psaltopoulou et al. (2013) observed that high adherence to a Mediterranean diet was associated with a reduced risk of cognitive impairment and depression, respectively. However, other studies have observed significant differences between cognitive performance and adherence to a Mediterranean diet (Chan, Chan & Woo, 2013; Samieri et al., 2013a; Samieri et al., 2013b), or demonstrated that this diet can improve cognitive performance in patients with no cognitive impairment. However, it remains unclear whether it can lower or postpone the risk of AD onset (Féart et al., 2009; Ngandu et al., 2015). In addition, a systematic review by Petersson & Philippou (2016) concluded that adherence to a Mediterranean diet was associated with improved cognitive performance, according to the findings of epidemiological studies. These findings underline the importance of good adherence to a Mediterranean diet. On the other hand, as we observed, no patient in our study showed high adherence to a Mediterranean diet.

The strengths of our study include the fact that it was carried out on patients who were being regularly followed up by an AD referral unit in our health region and therefore there was a high degree of diagnostic certainty. Another strength was that patients were assessed using different psychometric tests and nutritional scales, which allowed us to build up a global picture of the person’s general status and their eating habits. The study also had limitations because of it design. For example, the exclusion criteria included institutionalised patients and patients living at home with no reliable source of information about their eating habits. Patients who were at advanced stages of the disease and could not attend the unit were also excluded. Therefore, this study could not provide conclusions about the nutritional status of patients who had advanced stages of AD and limited support at home or about associated pathologies that carried the potential risk of anorexia, which may have lead to underestimating the prevalence of malnutrition found in people with AD.

## Conclusions

Our findings led us to conclude that there was a high prevalence of malnutrition and risk of malnutrition, and low adherence to a Mediterranean diet, in the patients diagnosed with AD in this study. In addition to behavioural alterations and caregiver’s overburden, the progression of AD was associated with worse nutritional status and correlated with cognition, daily activities and neuropsychiatric symptoms.

For all these reasons, we believe that it should be routine clinical practice for memory units to carry out appropriate evaluations of the nutritional status of patients diagnosed with AD. This would then enable clinical staff to provide support to ensure that their patients have a healthy nutritional status.

##  Supplemental Information

10.7717/peerj.5150/supp-1 Data S1 Raw data

## Additional Information and Declarations

### Data Availability

The following information was supplied regarding data availability: The raw data measurements and codes are provided in the Data S1.
